# Null functions in three-dimensional imaging of alpha and beta particles

**DOI:** 10.1038/s41598-017-16111-z

**Published:** 2017-11-17

**Authors:** Yijun Ding, Luca Caucci, Harrison H. Barrett

**Affiliations:** 1 Department of Physics, University of Arizona, Tucson, AZ USA; 2 Department of Medical Imaging, University of Arizona, Tucson, AZ USA; 3 College of Optical Sciences, University of Arizona, Tucson, AZ USA

## Abstract

Null functions of an imaging system are functions in the object space that give exactly zero data. Hence, they represent the intrinsic limitations of the imaging system. Null functions exist in all digital imaging systems, because these systems map continuous objects to discrete data. However, the emergence of detectors that measure continuous data, e.g. particle-processing (PP) detectors, has the potential to eliminate null functions. PP detectors process signals produced by each particle and estimate particle attributes, which include two position coordinates and three components of momentum, as continuous variables. We consider Charged-Particle Emission Tomography (CPET), which relies on data collected by a PP detector to reconstruct the 3D distribution of a radioisotope that emits alpha or beta particles, and show empirically that the null functions are significantly reduced for alpha particles if ≥3 attributes are measured or for beta particles with five attributes measured.

## Introduction

The performance of an imaging system is determined by the many physical processes involved. Imaging is ultimately about transferring information about the object to the detector. The information is often carried by photons and charged particles. The physical processes involved in photon and particle transport include emission, absorption, scattering, propagation and detection^1^. Each process affects the performance of an imaging system in a complicated way, but these effects in combination can be characterized by null functions.

Null functions represent the intrinsic limitation of a linear imaging system. A null function [ref.^2^, p. 34–38] is any non-zero function in the object space that produces no data in the data space. In other words, null functions are invisible to an imaging system because, if added to an object, any difference in the collected data is only due to random noise in the imaging system. For any linear system, an object can be decomposed into two components, the measurement component and the null component. The measurement component of an object provides a perfect fit to the noise-free data. The null component of an object, which is also referred to as a null function, makes no contribution to the data and cannot be recovered even from noise-free data.

Null functions exist in all digital imaging systems^3–9^, because real-world objects are functions of continuous variables while a digital image consists of an array of pixel values or other forms of histograms. The mapping from continuous objects to discrete data, which can be represented by a continuous-to-discrete (CD) operator, causes an infinite number of objects to produce exactly the same data. Therefore, null functions, which are the differences of those objects, are inevitable for a CD system.

Recent work^9^ has shown that null functions can be reduced if continuous data are collected instead of discrete data for Single Photon Emission Computed Tomography (SPECT). The continuous data are collected with photon-processing detectors^10–16^ that apply a maximum-likelihood^11,13,17–20^ method to estimate the interaction position, deposited energy and other attributes of each photon-interaction event.

Our previous work^21^ extended the concept of photon-processing detectors to particle-processing detectors that detect alpha and beta particles. Two main applications of alpha and beta particles are studying the metabolisms of pharmaceuticals and targeted radionuclide therapy for cancer. In both applications, the cellular-level distribution of the charged-particle-emitting radionuclide is valuable information that can be acquired through imaging. The conventional imaging technique for charged particles is autoradiography^22–26^, which provides two-dimensional, *ex vivo* images of thin tissue slices. In that paper^21^, we presented a direct three-dimensional autoradiography technique, which was named as charged-particle emission tomography (CPET). CPET was enabled by particle-processing detectors (PPDs).

A particle-processing detector (PPD) detects individual particles, estimates information about each particle, and stores the estimated particle parameters as continuous variables in a list^14–16,18,27^. On a detector plane, the interaction of a charged particle with the detector can be described by up to five parameters including two variables describing the interaction position, the deposited energy and two direction cosines describing the propagation direction. We refer to each parameter that describes the interaction event as an attribute of the detected particle. Upon interacting with a detector, each charged particle forms a track and generates secondaries along the track. The secondaries, which are photons for a scintillation detector and electron-hole pairs if a semiconductor detector is used, may contain information about a subset of the five particle attributes. From the raw signals produced by the secondaries, particle attributes can be estimated rigorously with maximum-likelihood estimation^11,13,17–20^. A few examples of PPDs were introduced in the main text and appendix of Ding, *et al*.^21^.

In this paper, with the goal of understanding the benefits of using a PPD, we calculate the null functions of CPET when different combinations of attributes are measured. Starting from Monte Carlo simulations or theoretical calculations of charged-particle transport, we use the Singular Value Decomposition (SVD) to calculate the null functions corresponding to a test object. Our results show that the null functions are significantly reduced for alpha particles with *q* ≥ 3 or for beta particles with *q* = 5, where *q* is the number of attributes estimated for each particle.

This paper is organized as follows. Section 1 introduces the setup and physics of CPET. Section 2 presents a mathematical model of the imaging system, which serves as a foundation for Section 3 and 4. Section 3 introduces the mathematical concept of null function. Section 4 presents detailed methods of this study. The results are discussed in Section 5. Section 6 provides a summary and directions for future work.

## Setup and physics of Charged-Particle Emission Tomography

Radioisotopes that emit charged particles, such as alpha and beta particles, are widely used to label molecules or cells of interest in pharmacology^23–26^ and targeted radiation therapy^28–31^. Imaging of tissue samples containing such radioisotopes provides information about the distribution of a pharmaceutical or radiation dose. In the conventional charged-particle imaging technique, which is autoradiography, if cellular-level resolution is desired, one has to slice the tissue samples to 4- to 10-*μ*m thick. This leads to a large number of tissue samples needed to be imaged and limits autoradiography to *ex vivo* imaging.

A direct three-dimensional imaging technique for radioisotopes that emit alpha or beta particles, Charged-Particle Emission Tomography (CPET), has been presented before^21,32,33^. CPET would allow imaging of thick tissue sections with high spatial resolution, which reduces the number of tissue samples needed for imaging. CPET also has the potential to be used for *in vivo* imaging. CPET is enabled by particle-processing detectors (PPDs)^21^. A PPD detects single particles and measures a subset of particle attributes, such as position, direction, and energy, for each detected particle.

**Figure 1:**
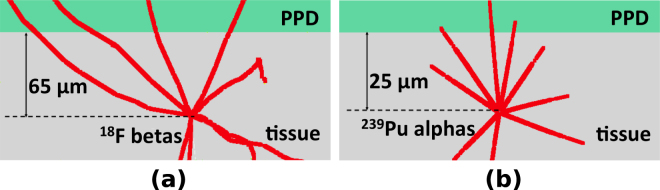
llustration of CPET systems. (**a**) BET system and Geant4-simulated^42,43^ tracks of positrons emitted from a ^18^F-point source in a 100-μm-thick tissue. (**b**) *α*ET and Geant4-simulated tracks of alpha particles emitted from a ^239^Pu-point source in a 40-μm-thick tissue.

In CPET^21^, the objects imaged are molecules or cells that have been labeled with charged-particle-emitting radioisotopes in tissue; a planar PPD is placed in contact with or in close proximity to one side of a sample tissue, as illustrated in Fig. 1. Beta Emission Tomography (BET) and Alpha Emission Tomography (αET, pronounced as *AlphET*) are two special cases of CPET. In each case, the detected-particle attributes are fed into an algorithm to reconstruct the 3D distribution of the radioisotopes. There is no motion of the detector or the object, making CPET a case of extreme limited-angle tomography^34^.

BET is an imaging technique for fast electrons, which include beta particles, conversion electrons and Auger electrons. Beta particles have broad, continuous energy spectra, while conversion electrons and Auger electrons usually have energy spectra with several sharp peaks. While traversing media, fast electrons deflect easily due to their low mass, and therefore fast electrons tend to have tortuous paths^35^. Figure 1(a) shows the simulated 3D beta particle tracks projected on a 2D plane, where the particles are emitted from a ^18^F-point source.

*α*ET produces images of radioisotopes that emit alpha particles^21,36^. Alpha particles have discrete energy spectra with highly monoenergetic emission lines associated with particular nuclear transitions. In low-atomic-number materials such as water or tissue, alpha particles travel in nearly straight lines because Rutherford scattering rarely happens^35^. As alpha particles travel through matter, they interact with the molecules in the material through Coulomb interactions and lose energy continuously. The energy loss of an alpha particle in a material is a known function of its path length. At the end of its path, an alpha particle loses all of its energy and is stopped in the material. Figure 1(b) shows the simulated 3D alpha particle tracks projected on a 2D plane, where the particles originate from a ^239^Pu-point source.

Due to the short range of alpha particles and significant scattering of beta particles in matter, CPET is designed differently from conventional emission tomography techniques such as Positron Emission Tomography (PET) and Single-Photon Emission Computed Tomography (SPECT), both of which collect 2D projections of the 3D object from multiple angles^37^. In contrast, CPET achieves tomographic reconstruction from a single projection data. Another fundamental difference between CPET and the conventional emission tomography (PET and SPECT) is that charged particles cannot penetrate the body of a patient unless the source is close to the skin. Potential applications of CPET in medicine include 3D imaging of superficial lesions, endoscopic tomography, surgical probes and 3D molecular imaging of a small animal with a window chamber.

## Mathematical Model of CPET

Rigorous analysis of CPET requires a mathematical model, which we derive step by step in this section. For an example on how to apply this mathematical model, see Section 4. In CPET, the object is the spatial density of radioactive decays per unit time. We denote the object as a scalar-valued function *f* **(R**), where **R** = (*x*, *y*, *z*) is a point within the object. We assume that the object is square-integrable and supported within a region **S**_*f*_ in ${{\mathbb{R}}}^{3}$. Therefore, the object function *f*(**R**), which is also referred to as **f** in the following discussion, is a vector in the Hilbert space ${{\mathbb{L}}}_{2}({{\bf{S}}}_{f})$.

The charged particles produced by the object propagate through tissue, and some of them enter the planar detector and produce signals. Each detected particle can be characterized by an attribute vector (**r**_*d*_, ***s***, *E*), where **r**_*d*_ = (*x*_*d*_, *y*_*d*_) is the 2D position of the particle at the detector plane, **s** = (*s*_*x*_, *s*_*y*_) is the direction cosines representing the propagation direction, and *E* is the residual energy. A statistical ensemble of particles is described by a phase-space distribution function, which is referred to as the radiance in the imaging literature^1,2^. The radiance of the charged-particles at the entrance plane of the detector is related to the object through

1 $${L}_{0}({{\bf{r}}}_{d},{\bf{s}},E)={\mathscr{P}}\,{\bf{f}},$$

where ${\mathscr{P}}$ is a propagation operator describing the interaction between the charged particles and tissue. The kernel of ${\mathscr{P}}$ is pr (**r**_*d*_, **s**, *E*|**R**)*S*(**R**), where pr(**r**_*d*_, **s**, *E*|**R**) is the probability density of a detected particle with true attribute vector (**r**_*d*_, **s**, *E*) given that the event originated with an emission at **R**; and *S*(**R**) is the probability that an emission at **R** will be detected and result in a list entry. The function *S*(**R**) is often referred to as the sensitivity function. Operator ${\mathscr{P}}$, which includes the physical processes of scattering and energy loss, can be derived analytically or approximated by Monte Carlo simulation.

A PPD collects signals from each detected particle and estimates a subset of the five attributes (*x*_*d*_, *y*_*d*_, *s*_*x*_, *s*_*y*_, *E*) to some uncertainty. The estimated attribute vector, which is a vector in the *q*-D attribute space (*q* = 2, 3, 4, 5), is denoted as $\hat{{\bf{A}}}$. If a total of *J* particles are detected, the output of a PPD is a list of *J* attribute vectors. We will denote this list as ${\mathscr{A}}=\{{\hat{{\bf{A}}}}_{1},{\hat{{\bf{A}}}}_{2},\ldots ,{\hat{{\bf{A}}}}_{J}\}$, where $\hat{{\bf{A}}}{j}$ is the estimated attribute vector for the *j* ^*th*^ detected event. Because the radioactive decays are independent events, the following Poisson point process [ref.^2^, p. 649–669] can be used to describe the list-mode data^15,27,38,39^

2 $$u(\hat{{\bf{A}}})=\sum _{j\mathrm{=1}}^{J}\delta (\hat{{\bf{A}}}-{\hat{{\bf{A}}}}_{j}),$$

where *δ*(...) denotes the *q*-D Dirac delta function. For a given object and a fixed exposure time *τ*, the mean of $u(\hat{{\bf{A}}})$ over an ensemble of attribute lists (more specifically, over the number of events *J* and each attribute vector ${\hat{{\bf{A}}}}_{j}$) is^27,32^

3 $$\begin{array}{ccc}\bar{u}(\hat{{\bf{A}}}|{\rm{f}}) & = & \sum _{J=0}^{+{\rm{\infty }}}Pr(J)\int {d}^{q}{\hat{{\bf{A}}}}_{1}\ldots \,\int {d}^{q}{\hat{{\bf{A}}}}_{J}\,{\rm{p}}{\rm{r}}(\hat{{\bf{A}}}|J)\sum _{j=0}^{J}\delta (\hat{{\bf{A}}}-{\hat{{\bf{A}}}}_{j})\\  & = & \tau {\int }_{{S}_{f}}{d}^{3}R\,f({\bf{R}})\,{\rm{p}}{\rm{r}}{\rm{f}}(\hat{{\bf{A}}}|{\bf{R}}),\end{array}$$

where ${\rm{prf}}(\hat{{\bf{A}}}|{\bf{R}})$ is the *point response function* with the point source located at **R**.

By measuring the attribute vector for each detected particle, the detector maps the phase-space distribution function *L*_0_(**r**_*d*_, **s**, *E*) to data $\bar{u}(\hat{{\bf{A}}}|{\bf{f}})$. This detection process can be described by a measurement operator ${\mathcal M}$ as

4 $$\bar{u}(\hat{{\bf{A}}}|{\bf{f}})= {\mathcal M} {L}_{0}({{\bf{r}}}_{d},{\bf{s}},E),$$

The imaging system can be described by combining Equation (1) and Equation (4):

5 $$\bar{{\bf{u}}}= {\mathcal M} {\mathscr{P}}{\bf{f}}= {\mathcal L} {\bf{f}}.$$

where ${\mathcal L} = {\mathcal M} {\mathscr{P}}$ is the system operator, and ${\bf{u}}=\bar{u}(\hat{{\bf{A}}}|{\bf{f}})$ is a vector in the Hilbert space ${{\mathbb{L}}}_{2}({{\mathbb{R}}}^{q})$. Thus, ${\mathcal L}$ is a linear operator that maps a vector **f** in ${{\mathbb{L}}}_{2}({{\bf{S}}}_{f})$ (object space) to a vector **u** in ${{\mathbb{L}}}_{2}({{\mathbb{R}}}^{q})$ (image space).

As shown in Equation (3), the kernel function of ${\mathcal L}$ is ${\rm{prf}}(\hat{{\bf{A}}}|{\bf{R}})$. This kernel function is given by

6 $${\rm{prf}}(\hat{{\bf{A}}}|{\bf{R}})=\int {d}^{q}{A}\,{\rm{pr}}(\hat{{\bf{A}}}|{\bf{A}})\,{\rm{pr}}({\bf{A}}|{\bf{R}})S({\bf{R}}),$$

where pr(**A**|**R**)**S**(**R**) is obtained by marginalizing pr(**r**_*d*_, **s**,*E*|**R**)*S*(**R**) over the particle attributes that are not estimated by the detector; and pr$(\hat{{\bf{A}}}|{\bf{A}})$ is a probability density function of the estimates $\hat{{\bf{A}}}$ when the true underlying attribute is **A**. The estimation uncertainty originates from noise in the detector.

## SVD and null functions

Null function analysis is based on Singular Value Decomposition (SVD). In this section, we introduce SVD and show how to use it to calculate null functions. In addition, we discuss the symmetries of CPET and how to apply symmetries to simplify the SVD analysis.

### Singular Value Decomposition

SVD analysis of a linear system involves the system operator and its adjoint operator [ref.^2^, p. 17]. The adjoint operator of ${\mathcal L}$, denoted as ${ {\mathcal L} }^{\dagger }$, maps a function in the attribute space to a function in the object space. The adjoint operator is defined by

7 $${\langle {\bar{{\bf{u}}}}_{1}, {\mathcal L} {{\bf{f}}}_{2}\rangle }_{{{\mathbb{L}}}_{2}({{\mathbb{R}}}^{q})}={\langle { {\mathcal L} }^{\dagger }{{\bf{u}}}_{1},{{\bf{f}}}_{2}\rangle }_{{{\mathbb{L}}}_{2}({{\bf{S}}}_{f})}$$

where 〈.,.〉 is the inner product in Hilbert space ${{\mathbb{L}}}_{2}({{\bf{S}}}_{f})$ or ${{\mathbb{L}}}_{2}({{\mathbb{R}}}^{q})$. The adjoint operator ${\mathcal L}$ ^†^ acting on a point process $u(A)={\sum }_{j\mathrm{=1}}^{J}\delta (\hat{{\bf{A}}}-{\hat{{\bf{A}}}}_{j})$ is a function of **R** and it is given by

8 $$\,{ {\mathcal L} }^{\dagger }{\bf{u}}=\tau \sum _{j\mathrm{=1}}^{J}{\rm{prf}}({{\bf{A}}}_{j}|{\bf{R}}\mathrm{).}$$

SVD is performed by solving the eigenequation generated by two operators $\,{ {\mathcal L} }^{\dagger }\, {\mathcal L} \,$ and $\, {\mathcal L}  {\mathcal L} {}^{\dagger }$. The operators $\,{ {\mathcal L} }^{\dagger }\, {\mathcal L} \,$ and $\, {\mathcal L}  {\mathcal L} {}^{\dagger }$ are Hermitian and non-negative definite, which means the operators have real and non-negative eigenvalues. Furthermore, the two operators have the same eigenvalue spectra^2^. The operator $\,{ {\mathcal L} }^{\dagger }\, {\mathcal L} \,$ maps a function from the object space back to the object space; while $\, {\mathcal L}  {\mathcal L} {}^{\dagger }$ maps a function from image space back to the image space. In tomography literature, $\,{ {\mathcal L} }^{\dagger }\, {\mathcal L} \,$ is often referred to as the projection/backprojection operator. The eigenfunctions of $\,{ {\mathcal L} }^{\dagger }\, {\mathcal L} \,$ and $\, {\mathcal L}  {\mathcal L} {}^{\dagger }$ are in the object space and the image space, respectively.

We will denote the eigenfunction of $\,{ {\mathcal L} }^{\dagger }\, {\mathcal L} \,$ as *w*_*n*_(*x*, *y*, *z*), the eigenfunction of $\, {\mathcal L}  {\mathcal L} {}^{\dagger }$ as *v*_*n*_(**A**), and the corresponding eigenvalue as *μ*_*n*_, where *n* can be an integer, a continuous variable, or a combination of both. When an eigenfunction in the object space is propagated through the imaging system, the resulted function is the corresponding eigenfunction in image space. This can be written in abstract form as

9 $$[\, {\mathcal L} \,{{\bf{w}}}_{n}]=\sqrt{{\mu }_{n}}{{\bf{v}}}_{n},{\rm{and}}\,[\, {\mathcal L} {}^{\dagger }{{\bf{v}}}_{n}]=\sqrt{{\mu }_{n}}{{\bf{w}}}_{n},$$

where ${{\bf{w}}}_{n}={w}_{n}(x,y,z)$ is the *n* ^*th*^ eigenfunction in object space, ${{\bf{v}}}_{n}={{\bf{v}}}_{n}(\hat{{\bf{A}}})$ is the *n* ^*th*^ eigenfunction in image space, and *μ*_*n*_ is the *n* ^*th*^ eigenvalue of both $\, {\mathcal L}  {\mathcal L} {}^{\dagger }$ and $\,{ {\mathcal L} }^{\dagger }\, {\mathcal L} \,$. The value $\sqrt{{\mu }_{n}}$ is referred to as the *n* ^*th*^ singular value of ${\mathcal L}$.

### Calculation of null functions

For any linear system, an object can be decomposed into two orthogonal components, the measurement component and the null component. In this section, we introduce how to calculate null functions, which are represented by the null components of any objects^2,9^.

When *n* is discrete, we can use the eigenfunction in object space, **w**_*n*_, and the eigenvalues to calculate the measurement component of any object function **f** as

10 $${{\bf{f}}}_{meas}=\sum _{n\mathrm{=1}}^{R}{{\bf{w}}}_{n}{{\bf{w}}}_{n}^{\dagger }{\bf{f}},$$

where *R* is the rank^2^ of $\,{ {\mathcal L} }^{\dagger }\, {\mathcal L} \,$ and the summation is over *n*. If *n* is a continuous variable, the summation turns into an integration.

The null component, which does not contribute to the data, can be calculated by

11 $${{\bf{f}}}_{null}={\bf{f}}-{{\bf{f}}}_{meas}\mathrm{.}$$

The measurement component and null component lie in different subspaces of object space [^2^, p~34–44]. One can prove that the measurement component and the null component of the same object are orthogonal. In mathematical terms, the inner product of the two components is

12 $$({{\bf{f}}}_{null},{{\bf{f}}}_{meas})={\int }_{{{\bf{S}}}_{f}}{d}^{3}R\,{f}_{meas}({\bf{R}})\,{f}_{null}({\bf{R}})\,=\,0.$$

Singular-value decomposition of objects into their measurement and null components is a powerful technique to investigate the intrinsic limitations of imaging systems^4–9^. SVD analysis of an imaging system that maps a function in 3D object space to *q*-D data space can be complex due to the high dimensions. However, this complexity is reduced by the symmetry of CPET.

### Symmetries

The symmetry of CPET is contained in the propagation operator ${\mathscr{P}}$. The propagation operator has translational and rotational symmetries under two conditions: (1) the detector is large compared to the size of the object and the range of the charged particles, and (2) the medium where radioactive sources are located is uniform. Under the assumption that the measurement operator ${\mathcal M}$ does not break these symmetries, the system operator ${\mathcal L}$ has translational and rotational symmetries, which can be used to simplify the characterization of the imaging system.

In CPET, the kernel function of the projection/backprojection operator, $\,{ {\mathcal L} }^{\dagger }\, {\mathcal L} \,$, is

13 $$k({\bf{R}},{\bf{R}}\text{'})={\tau }^{2}{\int }_{{{\mathbb{R}}}^{q}}d\hat{{\bf{A}}}\,\mathrm{prf}(\hat{{\bf{A}}}|{\bf{R}})\,\mathrm{prf}(\hat{{\bf{A}}}|{\bf{R}}\text{'}),$$

where the point response function prf(**A**|**R**) has been defined in Equation (6).

As discussed in Section 7.2.10 in Barrett and Myers (2004)^2^, the translational symmetry on the *xy*-plane simplifies the kernel of $\,{ {\mathcal L} }^{\dagger }\, {\mathcal L} \,$:

14 $$[{ {\mathcal L} }^{\dagger } {\mathcal L} {\bf{f}}]({\bf{R}})={\int }_{0}^{{z}_{max}}dz^{\prime} {\int }_{\infty }{d}^{2}{\bf{r}}{\boldsymbol{^{\prime} }}\,k({\bf{r}}-{\bf{r}}{\boldsymbol{^{\prime} }},z,z^{\prime} )f({\bf{R}}^{\prime} ),$$

where **r** = (*x*, *y*), **R **= (*x*, *y*, *z*) and *z*_*max*_ is either the thickness of the tissue sample or the maximum-tissue thickness a particle can penetrate. Due to the lateral-shift invariance, the 2D-Fourier-basis functions, exp (−2*πi* ***ρ*** ⋅ **r**), are eigenfunctions associated with the transverse direction, and the 2D spatial frequency ***ρ*** serve as an index for the eigenvalue equations.

The eigenvalue equation of $\,{ {\mathcal L} }^{\dagger }\, {\mathcal L} \,$ turns into

15 $${\int }_{0}^{{z}_{max}}dz^{\prime} K({\boldsymbol{\rho }},z,z^{\prime} ){W}_{j}({\boldsymbol{\rho }},z^{\prime} )={\mu }_{{\boldsymbol{\rho }},j}{W}_{j}({\boldsymbol{\rho }},z),$$

where *K*(***ρ***, *z*, *z*′) is the 2D-Fourier transform of *k*(**r** − **r**′, *z*, *z*′) with respect to vector **r** − **r′**; *W*_*j*_(***ρ***, *z*)*exp*(2*πi* ***ρ*** ⋅ **r**) is an eigenfunction with indices ***ρ*** and *j*; *μ*_***ρ***,*j*_ is the corresponding eigenvalue; ***ρ*** is a continuous index and associated with the transverse directions; the index *j* is connected to the longitudinal direction.

Let us consider a fixed ***ρ*** for now. The function *K*(***ρ***, *z*, *z*′), which can also be denoted as *K*_***ρ***_(*z*, *z*′), is considered as a function of two variables *z* and *z*′. For a fixed ***ρ***, Equation (15) is an eigenvalue equation. The compactness of the linear operator defined by Equation (15) determines if the index *j* is discrete or continuous. An operator is compact if its kernel satisfies the Hilbert-Schmidt condition^2^, which in the case of Equation (15) assumes the form

16 $${\int }_{0}^{{z}_{max}}dz{\int }_{0}^{{z}_{max}}dz^{\prime} {|K({\boldsymbol{\rho }},z,z^{\prime} |}^{2} < {\rm{\infty }}.$$

When *K*(***ρ***, *z*, *z*′) is bounded and *z*_*max*_ is finite, the operator defined by the integration has a discrete spectrum and the index *j* is discrete. As a side note, the Hilbert-Schmidt condition is satisfied only in the *z* direction, but not on the *xy* plane. All CPET setups discussed in the following section have bounded *K*(***ρ***, *z*, *z*′) for a fixed ***ρ***, except for *α*ET with a detector that measures 5D data (*x*_*d*_, *y*_*d*_, *s*_*x*_, *s*_*y*_, *E*).

In numerical calculations, the 1D integration described in Equation (15) can be approximated with a matrix multiplication. If *N* different depths *z* are sampled when *K*(***ρ***, *z*, *z*′) is calculated, Equation (15) turns into an eigenvalue problem for an *N* × *N* matrix at each ***ρ***.

If a system also has rotational symmetry with respect to *z*-axis, the kernel *K*(***ρ***, *z*, *z*′) reduces to *K*(*ρ*, *z*, *z*′), where *ρ* is the magnitude of the 2D spatial frequency ***ρ***. As a result, for two frequencies, ***ρ***_1_ and ***ρ***_2_, the 1D eigen-analysis problem described in Equation (15) is the same if *ρ*_1_ = *ρ*_2_.

## Methods

We use SVD analysis to compare 2D-, 3D-, 4D- and 5D-detectors for both BET and *α*ET. The number of dimensions simply refers to the number of attributes measured by the detector. More specifically, the four combinations of attributes to be measured are:

- (*x*_*d*_, *y*_*d*_),
- (*x*_*d*_, *y*_d_, *E*),
- (*x*_*d*_, *y*_d_, *s*_*x*_, s_*y*_),
- (*x*_*d*_, *y*_d_, *s*_*x*_, s_*y*_, *E*),

where (*x*_*d*_, *y*_*d*_, *s*_*x*_, *s*_*y*_, *E*) have been introduced in Section 2. For each setup, *K*(*ρ*, *z*, *z*′) is calculated and an eigenvalue problem is solved at each *ρ*.

The function describing the interaction between charged particles and tissue, pr(**A**|**R**)*S*(**R**), is approximated with Monte Carlo simulation results for beta particles and solved analytically for alpha particles.

The estimation error of the detector, which is described by pr(**Â**|**A**), can be modeled asymptotically as a multivariate Gaussian distribution function with the inverse covariance matrix equals to Fisher Information Matrix^40,41^, if Maximum-Likelihood (ML) estimation is used^13,17–19,41^. The asymptotic properties of ML estimation are satisfied in the limit of a large number of secondaries (photoelectrons for scintillation detectors or electron-hole pairs for semiconductor detectors). However, in the present treatment, in order to explore the upper limit on how well the imaging technique can perform, we effectively “turn off” the uncertainties due to the estimation process in the detector. Mathematically speaking, we assume $\mathrm{pr}(\hat{{\bf{A}}}|{\bf{A}})=\delta (\hat{{\bf{A}}}-{\bf{A}})$.

### Monte Carlo simulations for BET systems

In this section, we consider an ^18^F source, because it is widely used in Positron Emission Tomography. Two other sources, an ^131^I source and a 400-keV-monoenergetic electron source, are considered in Appendix B.

We use the Monte Carlo simulation toolkit Geant4^42,43^ to simulate the passage of beta particles through tissue. Even though Monte Carlo simulation has intrinsic randomness, the uncertainties can be reduced by simulating a large number of particles.

For each type of detector, a point source is simulated at depth *z* in a layer of 100-μm tissue (approximated by water), where *z* belongs to {5, 10, ..., 100} μm. For each depth, the emission and propagation of 10^7^ beta particles are simulated. A detector of size 1024 × 1024 *μm* ^2^ is placed on the top surface of the tissue layer and measures *q* attributes from each particle that enters the detector. The output of each simulation is a list of particle attributes. To estimate $\mathrm{pr}(\hat{{\bf{A}}}|{\bf{R}})$, we bin the list-mode data into histograms. The dimensions of the bins are defined as: Δ*x* = Δ*y* = 4 μm, Δ*θ* = 2°, Δ*ϕ* = 2° and Δ*E* = 20 keV. The bin size is determined so that finer sampling rate does not produce significantly different results.

### Theoretical calculations for *α*ET systems

We approximate the tracks of alpha particles in tissue as straight lines and derive the point spread function of *α*ET analytically. The derivations are provided in Appendix C. We consider a ^239^Pu source, which emits alpha particles with energies 5.15 MeV (73 %), 5.14 MeV (15 %) and 5.11 MeV (10 %). We use a monoenergetic source that emits 5.15-MeV alpha particles as an approximation. The range of a 5.15-MeV-alpha particle in tissue (approximated by water) is 39.4 μm.

A detector of size 160 × 160 *μm* ^2^ is placed at the top surface of the tissue layer and measures *q* attributes from each particle that enters the detector from the tissue. A detailed discussion of *α*ET can be found in Ding *et al*.^21^. For each type of detector, a point source is considered at depth *z* in a layer of 40- μm tissue, where *z* can take any value in {1, 3,..., 39} μm. When *q* takes values in {2, 3, 4}, *K*(***ρ***, *z*, *z*′) is evaluated numerically for (*z*, *z*′) on a 20 × 20 grid. When a 5D detector is used (*q* = 5), the eigenvalue spectrum of the system can be calculated analytically. However, in order to compare to the numerical results for *q* = {2, 3, 4}, we consider the 5D *α*ET on the same discrete depth *z* used for the other detectors, *z* = {1, 3, ...39} μm.

## Results

**Figure 2:**
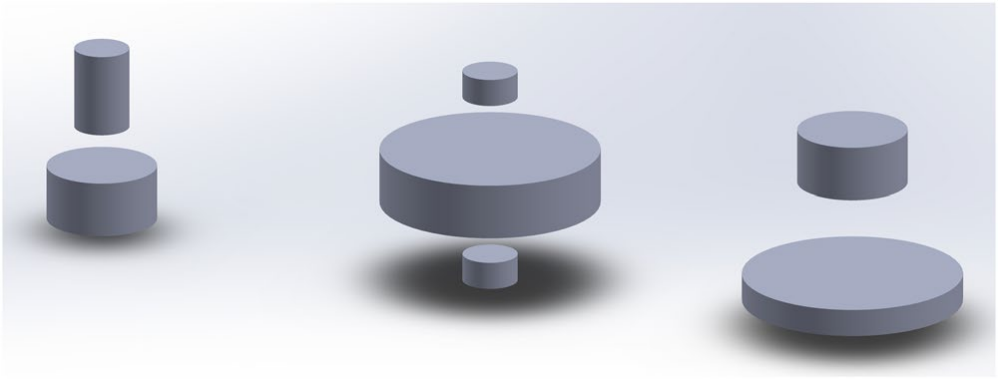
A 3D rendering of the simulated object in BET and *α*ET. The object, consisting of 7 cylinders of different sizes, is simulated in a layer of tissue. The tissue thickness is 100 μm for BET and 40 μm for *α*ET.

**Figure 3:**
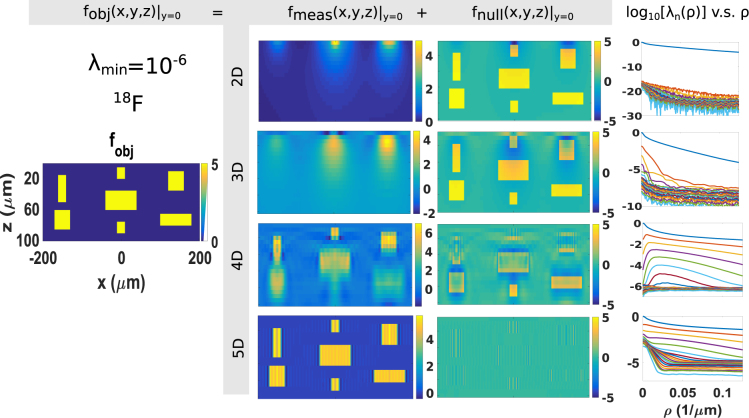
The coronal cross-section view (*xz*-plane) of the objects (Column 1), measurement components (Column 2) and null components (Column 3) and eigenvalue spectra in ^18^F BET systems with particle-processing detectors that measure 2D (Row 1), 3D (Row 2), 4D (Row 3) and 5D (Row 4) information about each detected particle, respectively. More specifically, 2D to 5D information refers to (*x*_*d*_, *y*_*d*_), (*x*_*d*_, *y*_*d*_, *E*), (*x*_*d*_, *y*_*d*_, *s*_*x*_, *s*_*y*_), and (*x*_*d*_, *y*_*d*_, *s*_*x*_, *s*_*y*_, *E*), respectively. The color scale represents radioactivity. The cutoff for *λ*_*n*_ is 10^−6^.

**Figure 4:**
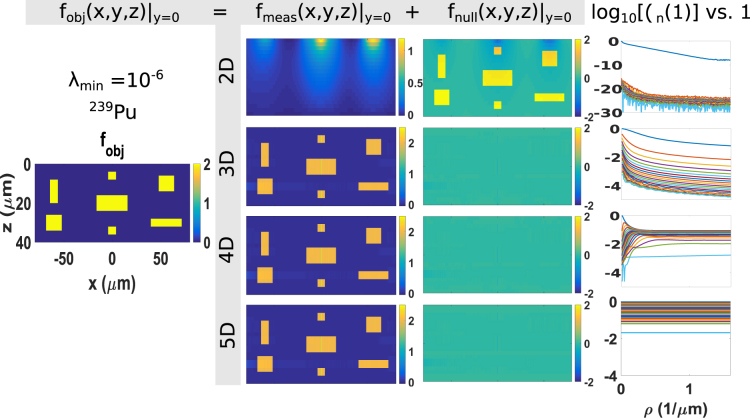
The coronal cross-section view (*xz*-plane) of the objects (Column 1), measurement components (Column 2) and null components (Column 3) and eigenvalue spectra in *α*ET with ^239^Pu sources, where particle-processing detectors that measure 2D (Row 1), 3D (Row 2), 4D (Row 3) and 5D (Row 4) information about each detected particle are used. More specifically, 2D to 5D information refers to (*x*_*d*_, *y*_*d*_), (*x*_*d*_, *y*_*d*_, *E*), (*x*_*d*_, *y*_*d*_, *s*_*x*_, *s*_*y*_), and (*x*_*d*_, *y*_*d*_, *s*_*x*_, *s*_*y*_, *E*), respectively. The color scale represents radioactivity. The cutoff for *λ*_*n*_ is 10^−6^.

In order to explore the intrinsic limitations in CPET, we calculate measurement and null components of a test object. We design an object **f** that contains several cylinders uniformly filled with radioactive substances. The size of the object scales to the tissue thickness of the system being studied (100 μm for BET and 40 μm for *α*ET). An illustration of the object, which shows the relative size, is provided in Fig. 2. For actual size of the objects in BET and *α*ET, see the cross-section views shown in column one of Figs 3 and 4, respectively.

The eigenvalue spectra and the eigenfunctions of $\,{ {\mathcal L} }^{\dagger }\, {\mathcal L} \,$ are calculated for each scenario. The eigenvalues of $\,{ {\mathcal L} }^{\dagger }\, {\mathcal L} \,$ are scaled to the maximum eigenvalue and presented in the fourth column of Figs 3 and 4 for BET and *α*ET, respectively. In the plots, the values *λ*_*n*_(*ρ*) = *μ*_*n*_(*ρ*)/*μ*_*max*_ are plotted as functions of spatial frequency *ρ*, where *μ*_*max*_ is the maximum eigenvalue. At each frequency, 20 eigenvalues are plotted with different colors.

The measurement component **f**_*meas*_ is calculated according to Equation 10, where the summation is for all *j* and ***ρ*** where *λ*_*j*,***ρ***_ > *λ*_*min*_. The threshold *λ*_*min*_ is chosen so that the eigenfunctions contributed to the measurement component are eigenfunctions with eigenvalues significantly above zero. As shown in the eigenvalue plots, *λ*_*j*,***ρ***_ are noisy (due to the numerical errors in the calculation of *K*(*ρ*, *z*, *z*′)) when *λ*_*j*,***ρ***_ < 10^−6^. Therefore, we set *λ*_*min*_ to 10^−6^. The results are shown for BET and *α*ET in Figs 3 and 4, respectively. Both figures present the object in the first column, the measurement component of the object in the second column, the null component in the third column. The four types of detectors that measure four different combinations of data are shown in rows one to four, respectively. All plots are *xz*-projection views of the 3D volume.

### BET

As shown from Row 1 to Row 4 in Fig. 3, when the number of measured attributes *q* increases from 2 to 5, the null component of the object reduces and the measurement component approaches the true object gradually. When all five attributes, (*x*_*d*_, *y*_*d*_, *s*_*x*_, *s*_*y*_, *E*), are measured from each detected particle, the null space of BET is almost completely eliminated. This means, despite the broad spectra of beta decays, it is necessary to measure the residual energy of the particles to achieve good depth resolution. For BET^21^, a preferred detector should have the ability to measure position, direction and energy of each detected particle.

### αET

The figures in the third column of Fig. 4 show that the null functions of *α*ET essentially disappear when the detector measures any of the following combinations of attributes (1) position and energy, (2) position and direction, and (3) position, direction and energy.

### Norm of the null functions

In order to quantitatively characterize the effect of measuring additional attributes on null functions, we calculate the scaled ${{\mathbb{L}}}_{2}$ norm of null functions. The ${{\mathbb{L}}}_{2}$ norm of **f** is defined as [ref.^2^, p.4]

17 $${\parallel {\bf{f}}\parallel }_{2}={[{\int }_{{{\bf{S}}}_{f}}{d}^{3}R{\bf{f}}{({\bf{R}})}^{2}]}^{\frac{1}{2}}.$$

We define the scaled norm of null functions as

18 $${\Vert {{\bf{f}}}_{null}\Vert }_{s}=\frac{{\Vert {{\bf{f}}}_{null}\Vert }_{2}}{{\Vert {\bf{f}}\Vert }_{2}},$$

where the subscript *s* represents scaled, and **f**_*null*_ is the null component of the object **f**.

**Figure 5:**
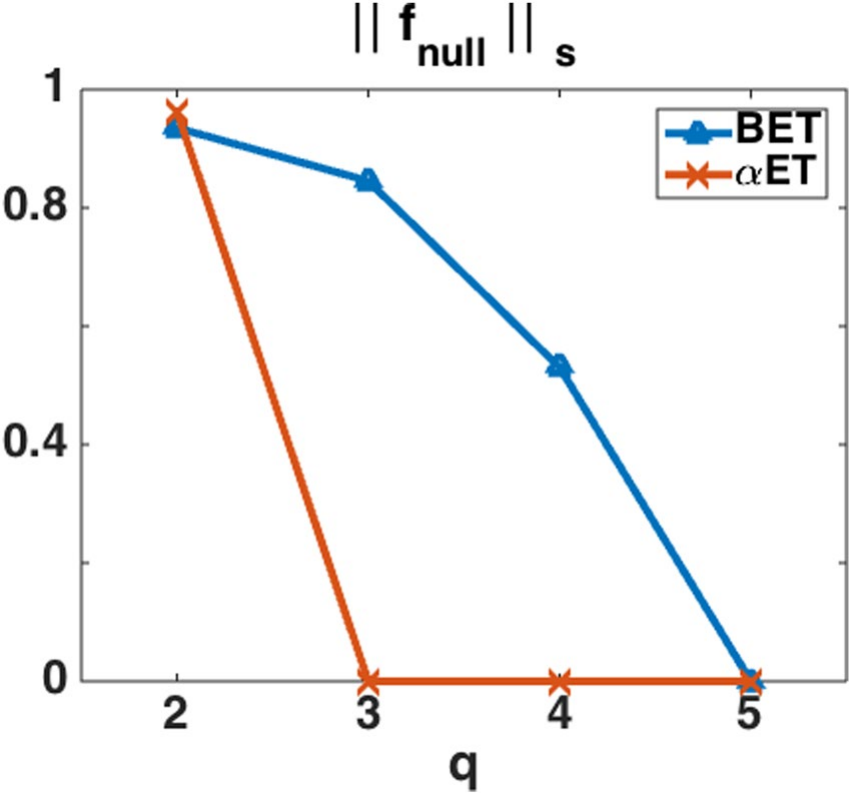
The norm of null functions vs. the number of measured attributes for BET and *α*ET, where ${\Vert {{\bf{f}}}_{null}\Vert }_{s}$ is the scaled norm of the null functions and *q* is the number of attributes estimated by the detector.

The scaled norm of null functions as a function of the number of attributes measured by the detector is plotted for both BET and *α*ET in Fig. 5. The plots show that as the number of attributes (*q*) measured by the detector increases, the scaled norm of the null functions decreases. The scaled norm of the null function goes to zero for *α*ET with *q* ≤ 3 and for BET with *q* = 5.

## Conclusions and future work

We have demonstrated the potential of particle-processing (PP) detectors in reducing null functions of Charged-Particle Emission Tomography (CPET). By collecting data with a PP detector, CPET is able to reconstruct the 3D distribution of a radioactive tracer that emits alpha or beta particles. Furthermore, this tomographic reconstruction is based on single-projection data. CPET is enabled by PP detectors that detect single particles and measure direction and energy attributes in addition to position for each particle.

Null functions are calculated for CPET for four different combinations of particle attributes. Our results show that, as the number of attributes measured increases, the null functions reduce for both alpha and beta particles. The direction attributes and the energy attribute, which are often ignored, contain important information for 3D reconstruction. The null functions of CPET are significantly reduced (1) for alpha particles when the direction or energy is measured along with the position for each particle and (2) for beta particles when the position, direction and energy are all measured for each detected particle.

In this paper, we studied the upper limits of the system performance by considering ideal detectors with no estimation uncertainty. For future work, the effect of estimation uncertainty on the null functions can be considered for a specific setup. Another avenue for future work includes studying charged-particle transport by solving the Boltzmann transport equation, which describes the temporal change of the radiance due to the physical processes of emission, scattering and energy loss. A numerical or analytical solution of the Boltzmann transport equation might provide more insights on beta emission tomography.

## Electronic supplementary material

Appendices A, B and C
