# Supplementary material for: Null functions in three-dimensional imaging of alpha and beta particles
Source: Sci Rep. 2017 Nov 17;7:15807. doi: 10.1038/s41598-017-16111-z (PMC5693958; doi:10.1038/s41598-017-16111-z)
Supplement: Supplementary file 1 — Appendices A, B and C [file 41598_2017_16111_MOESM1_ESM.pdf]

# Null functions in three-dimensional imaging of alpha and beta particles

**Yijun Ding**[1,*], **Luca Caucci**[2], **and Harrison H. Barrett**[2,3]

[1]Department of Physics, University of Arizona, Tucson, AZ
[2]Department of Medical Imaging, University of Arizona, Tucson, AZ
[3]College of Optical Sciences, University of Arizona, Tucson, AZ
[*]dingy@email.arizona.edu

## ABSTRACT

We supplement the main text with three appendices. Appendix A displays a few examples of eigenfunctions. Appendix B presents results on BET with $^{131}$I sources and mono-energetic 400-keV sources. Appendix C provides theoretical calculations for $\alpha$ET .

## A Eigenfunctions

We calculate a few 3D eigenfunctions of BET that measures 2D data $(x_d, y_d)$ and 5D data $(x_d, y_d, s_x, s_y, E)$, respectively. The $xz$-plane cross section of the eigenfunctions are presented in Figure 1.

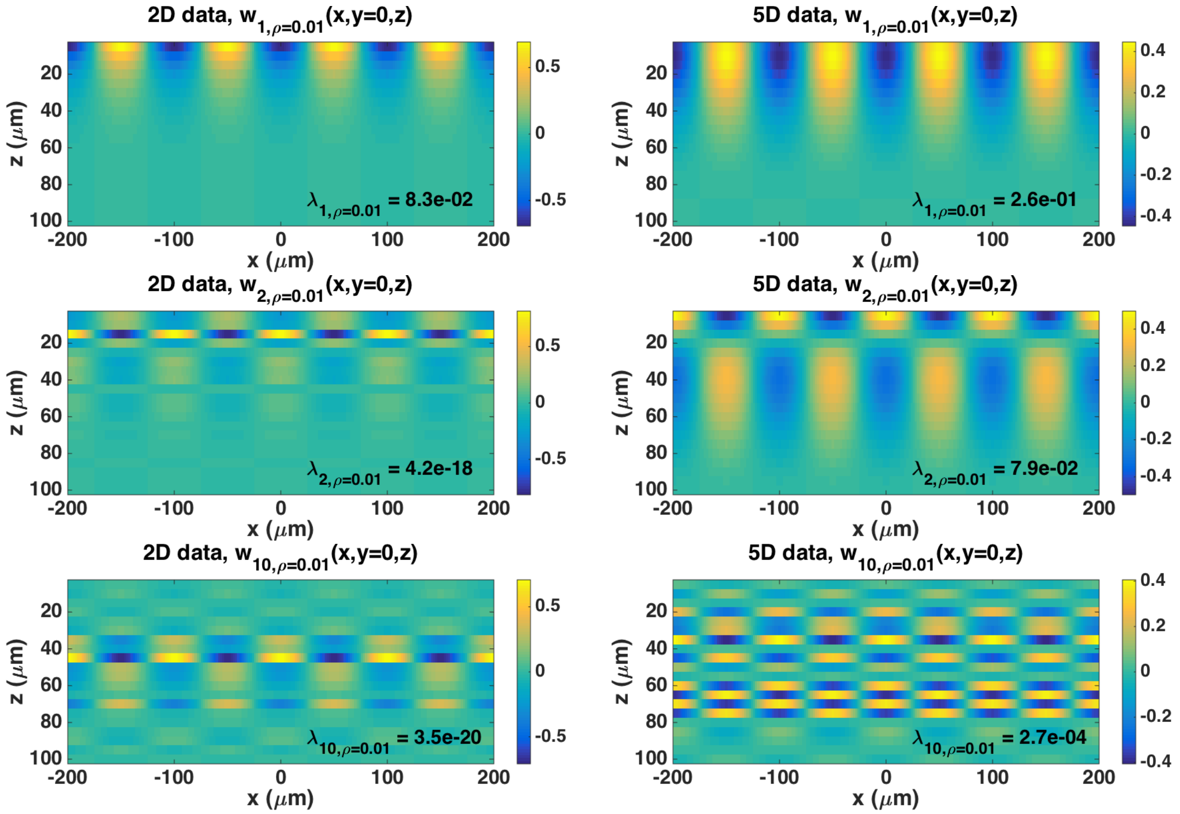


**Figure 1.** Cross-section view of 3D eigenfunctions in 2D BET (left) and 5D BET (right). The eigenfunctions are with indices $(\rho_x = 0.01, \rho_y = 0)\ \mu m^{-1}$, and $j = 1, 2, 10$ (Row 1, 2, 3, respectively). The scaled eigenvalues ($\lambda$, introduced in the main text[1]) corresponding to each eigenfunction are presented on the lower right corner of each plot. The plots show only the center of the object space.

## B Additional SVD analysis results of BET

In BET, structures of interest are labeled with fast-electron-emitting radionuclides; by imaging the fast electrons, information about the structure of interest can be reconstructed. Fast electrons include beta particles, conversion electrons and Auger electrons. Beta particles have broad energy spectra. Conversion electrons and Auger electrons have discrete energy spectra. For beta particles, the energy spectra of electrons and positrons are different due to the effect of the nuclear Coulomb field on the wave function of the beta particles.

We are interested in comparing the effects of different energy spectra on the performance of BET. For the comparison, we choose three sources which include $^{18}$F source, $^{131}$I source and a monoenergetic source that emits 400-keV electrons. The isotope $^{18}$F emits positrons (beta plus), $^{131}$I emits electrons (beta minus). The end-point energy of the two isotopes are similar. We choose the energy of the mono-energetic source at 400 keV, which is about 2/3 the end-point energy of $^{18}$F, for convenient comparison of the results obtained with different sources. The spectra of $^{18}$F and $^{131}$I are presented in Figure 2.

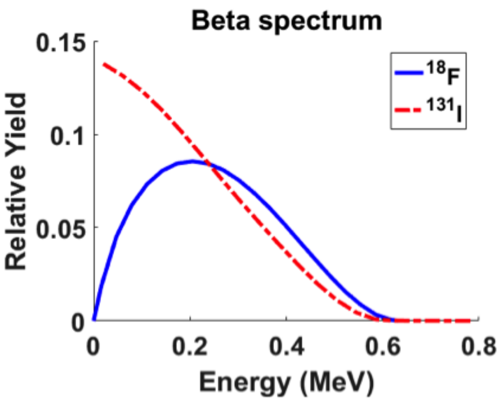


**Figure 2.** Spectra of F18 and I131.

The SVD analysis results of BET with an $^{18}$F source are presented in the main text[1]. In this supplement, we present SVD analysis results of BET with a 400-keV-monoenergetic source and BET with an $^{131}$I source. We use the same method, simulation parameters, bin size and test object presented in the paper for BET with $^{18}$F source.

The eigenfunctions that contributed to the measurement components corresponds to eigenvalues above a threshold. We set the threshold as $\lambda_{min} = 10^{-6}$. The results from BET with a source that emits 400-keV electrons are presented in Figure 3; and the results with the $^{131}$I source are presented in Figure 4.

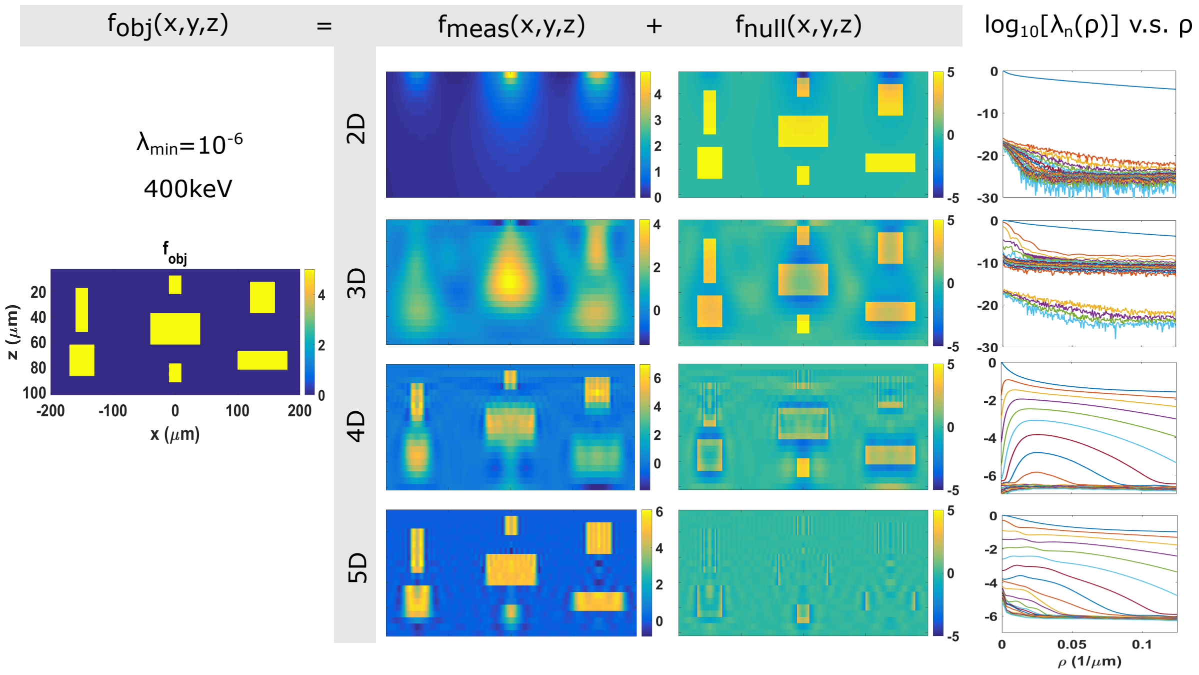


**Figure 3.** The coronal cross-section view ($xz$-plane) of the object (Column 1), measurement components (Column 2) and null components (Column 3) and eigenvalue spectra in BET systems where 400-keV mono-energetic electron sources are used. The results are with particle-processing detectors that measure 2D (Row 1), 3D (Row 2), 4D (Row 3) and 5D (Row 4) information about each detected particle, respectively. The color scale represents the radioactivity of the object. The cutoff for $\lambda_n$ is $10^{-6}$.

The results with the beta-minus source ($^{131}$I) and the mono-energetic source (400-keV) are similar to the results with

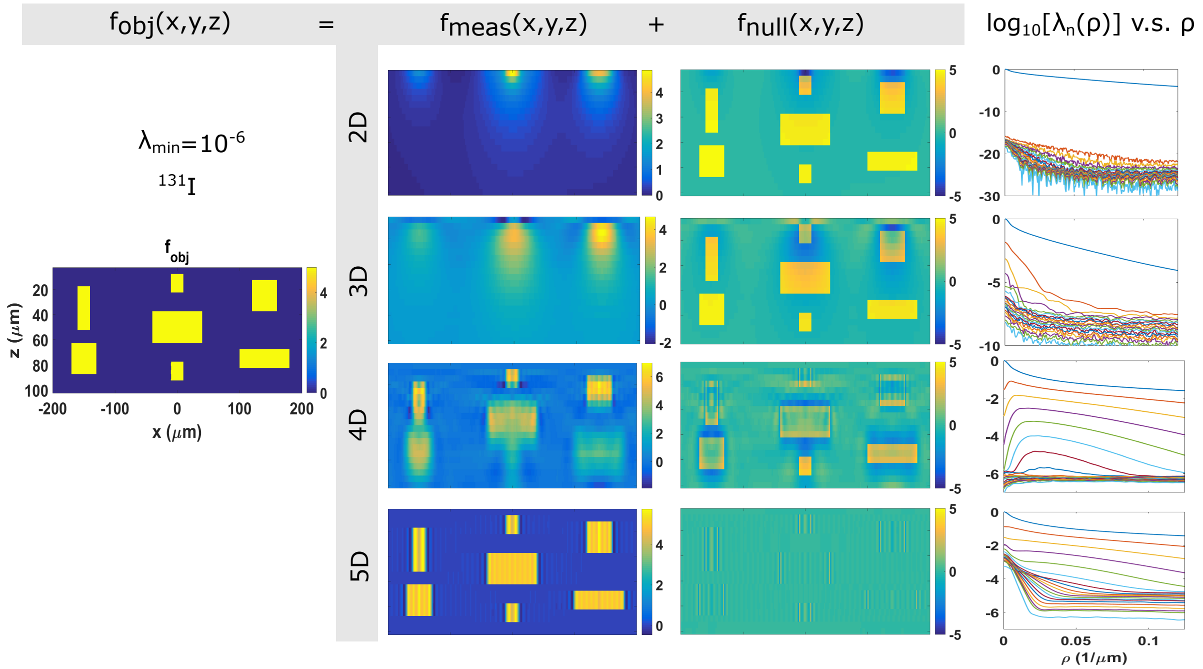


**Figure 4.** The coronal cross-section view ($xz$-plane) of the object (Column 1), measurement components (Column 2) and null components (Column 3) and eigenvalue spectra in $^{131}$I BET systems with particle-processing detectors that measure 2D (Row 1), 3D (Row 2), 4D (Row 3) and 5D (Row 4) information about each detected particle, respectively. The color scale represents the radioactivity of the object. The cutoff for $\lambda_n$ is $10^{-6}$. (Results of $^{18}$F are presented in the main text[1].)

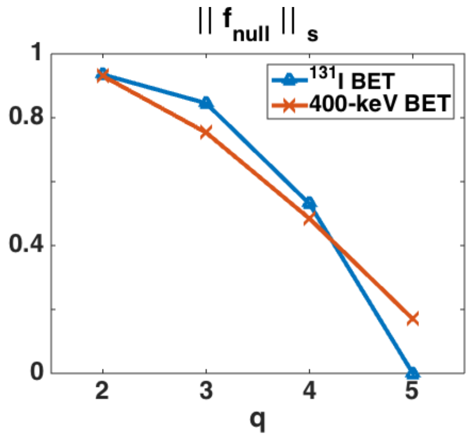


**Figure 5.** The scaled norm (introduced in the main text[1]) of null function as a function of the number of attributes measured by the detector. Results of BET with $^{131}$I source and BET with 400-keV mono-energetic source are presented. The symbol $||\mathbf{f}_{null}||_s$ is the scaled $\mathbb{L}_2$ norm of null functions. The symbol $q$ is the number of attributes measured by the detector.

beta-plus source ($^{18}$F) presented in the main text. As the number of attributes measured by the detector increases, the null space reduces. For BET with $^{131}$I source, when the detector measures 5D information, including detection position $(x_d, y_d)$, propagation direction $(s_x, s_y)$, and residual energy $E$, the null space is almost completely eliminated. For BET with the 400-keV mono-energetic source, when the detector measures 5D information, part of the object buried deeper in tissue is in the null space. That part of object cannot be recovered even with noise-free data. This might be due to the absence of particles with energy higher than 400-keV.

The scaled norm of the null functions ($||\mathbf{f}_{null}||_s$, introduced in the main text[1]) vs. the number of attributes measured by

the detector is plotted in Figure 5. The norm reduces as the number of measured attributes increases. When $q = 3$ or 4, the scaled norm of the null functions of BET with 400-keV mono-energetic sources is smaller than that with $^{131}I$ sources; but when $q = 5$, $||\mathbf{f}_{null}||_s$ for a 400-keV mono-energetic source is larger than that with a $^{131}I$ source. One possible explanation is that: the discreteness of the energy spectra of mono-energetic sources helps in reconstructing the source location when (1) position and energy or (2) position and direction are measured in BET; however, when position, energy and position are all measured for each detected particle, the maximum energy emitted by the source plays a bigger role in determining how well an object can be reconstructed.

In conclusion, the different shape in energy spectra from beta-minus decay and beta-plus decay does not lead to significantly different performance of BET with beta-minus source and beta-plus source. However, the particles emitted with higher energy contributes to the resolution deeper in tissue.

## C SVD analysis of $\alpha$ET

We present theoretical calculations for $\alpha$ET with 2D, 3D, 4D, and 5D detectors in this supplemental material.

From Equation (13) in the main text, the kernel function of $\mathcal{L}^\dagger\mathcal{L}$ follows

$$k(\mathbf{r}-\mathbf{r}', z, z') = \tau^2 \int d\hat{\mathbf{A}}\ \mathrm{prf}(\hat{\mathbf{A}}|\mathbf{r}, z)\ \mathrm{prf}(\hat{\mathbf{A}}|\mathbf{r}', z'), \tag{1}$$

where $\mathrm{prf}(\hat{\mathbf{A}}|\mathbf{r}, z)$ is the system response to a point source located at $(\mathbf{r}, z)$ and $\hat{\mathbf{A}}$ is a vector formed by the attributes measured by the system. The attributes includes detection position represented by $\hat{\mathbf{r}}_d = (\hat{x}_d, \hat{y}_d)$, propagation direction represented by $(\hat{s}_x, \hat{s}_y)$, and residual energy $\hat{E}$.

The symbol $\mathbf{A}$ represents a vector of the true underlying attributes; while $\hat{\mathbf{A}}$ represents a vector of the measured attributes, which contains detector estimation uncertainty. In this paper, we focus on the intrinsic limitation of $\alpha$ET systems and temporarily ignore the detector noise. Therefore, we use $\hat{\mathbf{A}}$ and $\mathbf{A}$ interchangeably in the following discussion.

### C.1 Point response functions

In a typical $\alpha$ET setup, alpha-particle-emitting sources are in a layer of tissue and a detector is placed at plane $z = 0$, as illustrated in Figure 6. Alpha particles travel in almost straight lines and lose energy continuously along their tracks[2]. The maximum distance a particle can travel in tissue, which we denote as $l_0$, is a function of the emission energy of the particle. With a point source, the alpha particles can be detected only in a circle determined by the source location and the emission energy[3,4].

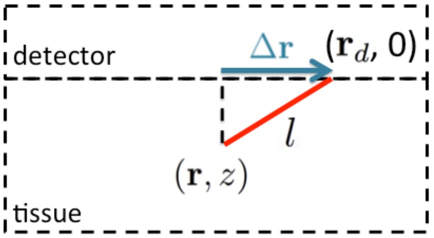


**Figure 6.** Illustration of the coordinate system of an $\alpha$ET system.

To describe the point response functions for $\alpha$ET systems, we assume the coordinate system as illustrated in Figure 6, where a particle is emitted from point $(\mathbf{r}, z)$ in tissue and detected at point $\mathbf{r}_d$ in the detector. We define the displacement between the detection location and the projection of the emission point on the detector plane as $\Delta\mathbf{r} = \mathbf{r}_d - \mathbf{r}$. We denote the distance from the emission position to the detection location is denoted as $l$, where $l = \sqrt{|\Delta\mathbf{r}|^2 + z^2}$ and $|\Delta\mathbf{r}|$ is the magnitude of the vector $\Delta\mathbf{r}$.

With this notation, the point response function $\mathrm{prf}(\mathbf{A}|\mathbf{r}, z)$ for $\alpha$ET with a particle-processing detector that measures $q$-D information for $q = \{2, 3, 4, 5\}$ are:

$$2D: \ \mathrm{prf}(\mathbf{r}_d|\mathbf{r}, z) = \frac{z}{4\pi l^3}\,\mathrm{cyl}\left(\frac{|\Delta\mathbf{r}|}{r_0(z)}\right)\mathrm{win}\left(\frac{z}{l_0}\right) \tag{2}$$

$$3D: \ \mathrm{prf}(\mathbf{r}_d, E|\mathbf{r}, z) = \frac{z}{4\pi l^3}\,\delta(E - E(l))\,\mathrm{cyl}\left(\frac{|\Delta\mathbf{r}|}{r_0(z)}\right)\mathrm{win}\left(\frac{z}{l_0}\right) \tag{3}$$

$$4D: \ \mathrm{prf}(\mathbf{r}_d, s_x, s_y|\mathbf{r}, z) = \frac{z}{4\pi l^3}\,\delta\left(s_x - \frac{x_d - x}{l}\right)\delta\left(s_y - \frac{y_d - y}{l}\right)\mathrm{cyl}\left(\frac{|\Delta\mathbf{r}|}{r_0(z)}\right)\mathrm{win}\left(\frac{z}{l_0}\right) \tag{4}$$

$$5D: \ \mathrm{prf}(\mathbf{r}_d, s_x, s_y, E|\mathbf{r}, z) = \frac{z}{4\pi l^3}\,\delta\left(s_x - \frac{x_d - x}{l}\right)\delta\left(s_y - \frac{y_d - y}{l}\right)\delta(E - E(l))\,\mathrm{cyl}\left(\frac{|\Delta\mathbf{r}|}{r_0(z)}\right)\mathrm{win}\left(\frac{z}{l_0}\right) \tag{5}$$

where $\mathrm{cyl}(r)$ is a *cylinder function* and $\mathrm{win}(t)$ is a *window function*. The cylinder function is unity inside a disc of radius 1 and zero outside:

$$\mathrm{cyl}(r) \equiv \begin{cases} 1\,, & \text{if } r < 1\,, \\ 0\,, & \text{if } r \geqslant 1\,. \end{cases} \tag{6}$$

The window function is defined by

$$\mathrm{win}(t) \equiv \begin{cases} 1\,, & \text{if } 0 \leqslant t < 1\,, \\ 0\,, & \text{if } t < 0 \text{ or } t \geqslant 1\,. \end{cases} \tag{7}$$

In the following discussion, we consider depth $z < l_0$, so that we can leave out the factor $\mathrm{win}(z/l_0)$ for now.

### C.2 2D detector

For a 2D detector, the 2D Fourier transform of a rotational symmetric function is the zero-th order Hankel transform[5]. The 2D Fourier transform of $z/4\pi l^3$ with respect to $\Delta\mathbf{r}$ is[6]

$$\mathcal{F}_2\left\{\frac{z}{4\pi l^3}\right\} = \frac{\exp(-2\pi z\rho)}{2}, \tag{8}$$

where $\rho$ is the magnitude of $\rho$. The 2D Fourier transform of a cylinder function is a *besinc* function[5],

$$\mathcal{F}_2\left\{\mathrm{cyl}\left(\frac{r_d}{r_z}\right)\right\} = \pi r_z^2\,\mathrm{besinc}(2\rho r_z). \tag{9}$$

The *besinc* function is defined as

$$\mathrm{besinc}(t) \equiv \frac{2J_1(\pi t)}{\pi t}, \tag{10}$$

where $J_1(\cdot)$ is the first-order Bessel function of the first kind[7]. The besinc function is also referred to as a jinc or sombrero function. For $\alpha$ET with a 2D detector, the 2D Fourier transform of $\mathrm{prf}(\mathbf{A}|\mathbf{r},z)$ with respect to $\Delta\mathbf{r}$ is

$$\mathrm{PRF}(\rho|z) = \left[\frac{\exp(-2\pi z\rho)}{2}\cdot\mathrm{win}\left(\frac{z}{l_0}-\frac{1}{2}\right)\right] * \left[\pi r_z^2\cdot\frac{2J_1(2\pi\rho r_z)}{2\pi\rho r_z}\right] \tag{11}$$

where the symbol $*$ represents convolution with respect to the 2D spatial frequency $\rho$.

The kernel function of $\mathcal{L}^\dagger\mathcal{L}$ is

$$k(\mathbf{r}-\mathbf{r}',z,z') = \tau^2\int d^2r_d\;\mathrm{prf}(\mathbf{r}_d|\mathbf{r},z)\;\mathrm{prf}(\mathbf{r}_d|\mathbf{r}',z'). \tag{12}$$

Since $\mathrm{prf}(\mathbf{r}_d|\mathbf{r},z) = \mathrm{prf}(\mathbf{r}_d-\mathbf{r}|z) = \mathrm{prf}(\mathbf{r}-\mathbf{r}_d|z)$, the kernel function is a convolution of the two point response functions. Therefore, the 2D Fourier transform of function $k(\mathbf{r}-\mathbf{r}',z,z')$ with respect to $(\mathbf{r}-\mathbf{r}')$ is

$$K(\rho,z,z') = \tau^2\mathrm{PRF}^*(\rho|z)\,\mathrm{PRF}(\rho|z'), \tag{13}$$

where the superscript $^*$ represents complex conjugate. The function $K(\rho,z,z')$ can be evaluated numerically.

### C.3 3D detector

With a 3D detector that measures position and energy $(x_d, y_d, E)$ of each detected particle, the kernel function of $\mathcal{L}^\dagger\mathcal{L}$ operator is

$$\begin{aligned} k(\mathbf{r}-\mathbf{r}',z,z') &= \tau^2\int_0^{E_{max}} dE\int_\infty d^2r_d\;\mathrm{prf}(\mathbf{r}_d-\mathbf{r},E|z)\;\mathrm{prf}(\mathbf{r}_d-\mathbf{r}',E|z') \\ &= \begin{cases} \tau^2\dfrac{zz'}{8\pi^2\,w(|\mathbf{r}-\mathbf{r}'|,z,z')}\left[\displaystyle\int_{l_{min}}^{l_0} dl\;\frac{1}{l^4\sqrt{l^2-l_{min}^2}}\left|\frac{dl}{dE}\right|\right]\mathrm{cyl}\left(\dfrac{l_{min}}{l_0}\right), & \text{if } \mathbf{r}=\mathbf{r}'; \\ \tau^2\dfrac{z\,\delta(z-z')}{8\pi}\displaystyle\int_z^{l_0} dl\;\frac{1}{l^4}\left|\frac{dl}{dE}\right|_l, & \text{if } \mathbf{r}\neq\mathbf{r}'; \end{cases} \end{aligned} \tag{14}$$

where

$$w(|\mathbf{r}-\mathbf{r}'|,z,z') = \frac{|(z^2 - z'^2 + |\mathbf{r}-\mathbf{r}'|^2)|}{2|\mathbf{r}-\mathbf{r}'|}, \tag{15}$$

and

$$l_{min} = \sqrt{w(|\mathbf{r}-\mathbf{r}'|,z,z')^2 + z'^2}. \tag{16}$$

We first evaluate $k(\mathbf{r}-\mathbf{r}',z,z')$ numerically, and then calculate $K(\rho,z,z')$ through Hankel transform.

### C.4 4D detector

With a 4D detector that measures position and direction $(x_d, y_d, s_x, s_y)$, the kernel function of $\mathcal{L}^\dagger\mathcal{L}$ operator is

$$\begin{aligned}
k(\mathbf{r}-\mathbf{r}',z,z') &= \tau^2 \int d\Omega \int_\infty d^2 r_d \,\mathrm{prf}(\mathbf{r}_d - \mathbf{r}, E|z)\,\mathrm{prf}(\mathbf{r}_d - \mathbf{r}', E|z') \\
&= \begin{cases} \tau^2 \dfrac{1}{16\pi^2} \dfrac{(z-z')^2}{[|\mathbf{r}-\mathbf{r}'|^2 + (z-z')^2]^2} \,\mathrm{cyl}\left(\dfrac{z'|\mathbf{r}-\mathbf{r}'|}{\sqrt{(l_0^2 - z'^2)(z-z')^2}}\right) \mathrm{cyl}\left(\dfrac{z|\mathbf{r}-\mathbf{r}'|}{\sqrt{(l_0^2 - z^2)(z-z')^2}}\right), & \text{if } z \neq z'; \\[2ex] \tau^2 \dfrac{\delta(\mathbf{r}-\mathbf{r}')}{16\pi} \left(1 - \dfrac{z^2}{l_0^2}\right), & \text{if } z = z'. \end{cases}
\end{aligned} \tag{17}$$

We can evaluate $k(\mathbf{r}-\mathbf{r}',z,z')$ numerically, and then calculate $K(\rho,z,z')$ through Hankel transform.

### C.5 5D detector

With a 5D detector that measures position and direction $(x_d, y_d, s_x, s_y, E)$, the kernel function of $\mathcal{L}^\dagger\mathcal{L}$ operator is

$$\begin{aligned}
k(\mathbf{r}-\mathbf{r}',z,z') &= \tau^2 \int d\Omega \int_0^{E_{max}} dE \int_\infty d^2 r_d \,\mathrm{prf}(\mathbf{r}_d - \mathbf{r}, E|z)\,\mathrm{prf}(\mathbf{r}_d - \mathbf{r}', E|z') \\
&= \tau^2\, \delta(\mathbf{r}-\mathbf{r}')\delta(z-z') \frac{z}{8\pi} \int_z^{l_0} \frac{dl}{l^2} \left|\frac{dl}{dE}\right|_l \mathrm{win}\left(\frac{z}{l_0}\right) \\
&= \tau^2\, \delta(\mathbf{r}-\mathbf{r}')\delta(z-z')\, p(z)\, \mathrm{win}\left(\frac{z}{l_0}\right)
\end{aligned} \tag{18}$$

The function $p(z)$ is defined as

$$p(z) \equiv \frac{z}{8\pi} \int_z^{l_0} \frac{dl}{l^2} \left|\frac{dl}{dE}\right|_l, \tag{19}$$

where $l_0$ and $dl/dE$ are determined by the emission energy of the particle and the material which the particle traveled in. For $^{239}$Pu alphas in tissue, $p(z)$ is plotted in Figure 7.

From Equation (18), we can write down

$$[\mathcal{L}^\dagger\mathcal{L}\mathbf{f}](x,y,z) = \int d^3R\, \tau^2\, \delta(\mathbf{r}-\mathbf{r}')\delta(z-z') f(x',y',z')\, p(z,l_0)\, \mathrm{win}\left(\frac{z}{l_0}\right) \tag{20}$$

$$= \tau^2 f(x,y,z)\, p(z)\, \mathrm{win}\left(\frac{z}{l_0}\right). \tag{21}$$

The eigenfunction of $\mathcal{L}^\dagger\mathcal{L}$ with a 5D detector is $u(x,y,z) = t(x,y)\cdot\delta(z-z_0)$, where $t(x,y)$ is any function, and the corresponding eigenvalue is $\tau^2 p(z_0)\mathrm{win}(z_0/l_0)$. Note the plot in Figure 7 is the eigenvalue spectrum of $\mathcal{L}^\dagger\mathcal{L}$ scaled by $1/\tau^2$. The measurement component of any object $f(x,y,z)$ is $f(x,y,z)\mathrm{win}(z/l_0)$.

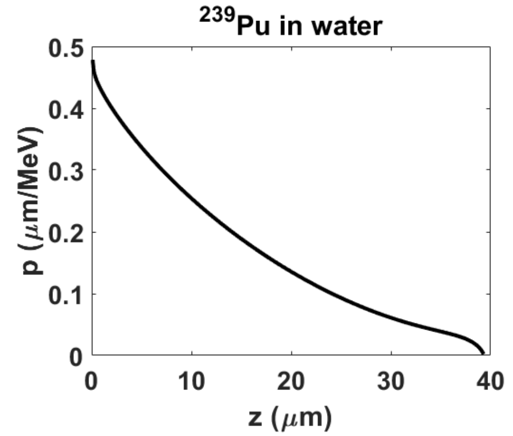


**Figure 7.** The factor p(z) for $^{239}$Pu $\alpha$ET with a detector that is able to measure 5 attributes, $(x_d, y_d, s_x, s_y, E)$, from each detection event.

95 ## References


**1.** Ding, Y., Caucci, L. & Barrett, H. Null functions in three-dimensional imaging of alpha and beta particles. *Scientific Reports* (2017).

**2.** Knoll, G. F. *Radiation detection and measurement* (John Wiley & Sons, Hoboken, NJ, 2010).

**3.** Ding, Y., Caucci, L. & Barrett, H. $\alpha$ET : Alpha Emission Tomography. *IEEE Nuclear Science Symposium and Medical Imaging Conference (NSS/MIC)* 1–3 (2014).

**4.** Ding, Y., Caucci, L. & Barrett, H. H. Charged-particle emission tomography. *Medical Physics* **44**, 2478–2489 (2017).

**5.** Barrett, H. H. & Myers, K. J. *Foundations of Image Science* (John Wiley & Sons, Hoboken, NJ, 2004).

**6.** Bracewell, R. N. *The Fourier Transform and Its Applications* (McGraw-Hill, 1999).

**7.** Abramowitz, M. & Stegun, I. *Handbook of Mathematical Functions* (Dover, New York, 1964), fifth edn.
